# Administration of *Bifidobacterium bifidum* BGN4 and *Bifidobacterium longum* BORI Improves Cognitive and Memory Function in the Mouse Model of Alzheimer’s Disease

**DOI:** 10.3389/fnagi.2021.709091

**Published:** 2021-08-06

**Authors:** Hongwon Kim, Sumin Kim, Sang-jun Park, Gwoncheol Park, Hakdong Shin, Myeong Soo Park, Jongpil Kim

**Affiliations:** ^1^Department of Biomedical Engineering, Laboratory of Stem Cells and Cell Reprogramming, Dongguk University, Seoul, South Korea; ^2^Department of Chemistry, Dongguk University, Seoul, South Korea; ^3^Research Center, BIFIDO Co., Ltd, Hongcheon, South Korea; ^4^Department of Food Science and Biotechnology, Sejong University, Seoul, South Korea

**Keywords:** Alzheimer’s disease, probiotics, gut microbiota, *Bifidobacterium*, cognitive and memory impairment

## Abstract

Recent evidence indicates that gut microbiota could interact with the central nervous system and affect brain function, including cognition and memory. In this study, we investigated whether *Bifidobacterium bifidum* BGN4 (*B. bifidum* BGN4)* and Bifidobacterium longum* BORI (*B. longum* BORI) alleviated the pathological features in a mouse model of Alzheimer’s disease (AD). Administration of *B. bifidum* BGN4 and *B. longum* BORI effectively suppressed amyloidosis and apoptotic processes and improved synaptic plasticity by ameliorating the neuroinflammatory response and BDNF expression. Moreover, behavioral tests indicated that *B. bifidum* BGN4 and *B. longum* BORI attenuated the cognitive and memory disability of AD mice. Taken together, the present study highlights the therapeutic potential of *B. bifidum* BGN4 and *B. longum* BORI for suppressing the pathological features of AD.

## Introduction

Alzheimer’s disease (AD) is a progressive neurodegenerative disorder that causes cognitive decline and memory loss. Its major pathological hallmarks are an accumulation of amyloid peptides, which are products of amyloid precursor protein (APP), and intracellular neurofibrillary tangles of hyperphosphorylated tau protein (Jucker and Walker, 2011). Mutations in APP, presenilin, and tau genes cause the development of relevant molecular pathologies inside neurons, leading to neuroinflammation and altered synaptic plasticity, and eventually resulting in neuronal death (Goate et al., 1991; Wolfe et al., 1999). Previous studies have shown that alternative therapies, including acupuncture, meditation, and medical foods, can treat AD (Thaipisuttikul and Galvin, 2012; Jia et al., 2017; Innes et al., 2018). Additionally, dietary supplements, such as unsaturated fatty acids, and vegetables, legumes, fruits, and omega prevent AD by reducing LDL cholesterol levels, have antioxidant and anti-inflammatory effects, and attenuate cognitive decline (Hu et al., 2013; El-Sayyad, 2015; Mendiola-Precoma et al., 2016).

Recently, studies have implicated gut microbiota in several neurological disorders, such as AD (Vogt et al., 2017), autism spectrum disorder (Sgritta et al., 2019), and Parkinson’s disease (Lee et al., 2019). Based on the concept of the microbiota-gut-axis, the relationship between the CNS and gut microbiota contributes a key role in disease risk, and the activity and progression of neurological disorders. For example, the gut microbiota, including Ruminococcaceae, Rikenellaceae, Clostridiaceae, and Enterobacteriaceae, acts as key regulators of neuroinflammation in aged mice (Conley et al., 2016; Matt et al., 2018). Moreover, an increase in the abundance of the pro-inflammatory gut microbiota taxon, Escherichia/Shigella, accelerates brain amyloidosis in cognitively impaired elderly individuals (Cattaneo et al., 2017). Moreover, many studies showed that the altered gut microbiota secretes immunogenic compounds such as amyloid, lipopolysaccharides (LPS), and other microbial exudates which mediate the effects of microbiota in several neurological disorders. For example, LPS can be translocated from the gut to the brain, exacerbating amyloid deposition in the AD model (Zhan et al., 2016). Sgritta et al. (2019) also reported that alteration of *L. reuteri* in gut microbiota modulates the expression of oxytocin *via* the vagus nerve in mouse models of autism spectrum disorder (Sgritta et al., 2019). Additionally, truncal vagotormy in mice prevented the spread of α-synucleinopathy from the gut to the brain (Kim et al., 2019), indicating that these immunogenic compounds mediate afferent and efferent pathways such as the vagus nerve or the hypothalamic-pituitary adrenal pathway (Grenham et al., 2011; Sgritta et al., 2019). Taken together, these results suggest that alterations in gut microbiota composition by probiotics may provide a novel therapeutic approach or ameliorating strategy for AD. Consistent with these results, recent studies have shown that the treatment of *Bifidobacterium breve* strain A1 alters gut microbiota and ameliorates cognitive dysfunction in an Aβ-induced AD mouse model (Kobayashi et al., 2017). In addition, the administration of xylooligosaccharides attenuates surgery-induced cognitive dysfunction in APP/PS1 AD mice by altering intestinal microbiota (Han et al., 2020). Moreover, in one of our previous our study, probiotic supplementation containing *B. bifidum* BGN4 and *B. longum* BORI was shown to improve mental flexibility and alleviate stress in healthy older adults (Kim et al., 2021). Therefore, from this perspective, we asked whether probiotics containing *B. bifidum* BGN4 and *B. longum* BORI can alleviate AD pathologies, such as cognitive dysfunction and memory loss.

In this study, in order to examine the therapeutic effects of *B. bifidum* BGN4 and *B. longum* BORI administration for AD, probiotic supplementation containing *B. bifidum* BGN4 and *B. longum* BORI was treated by oral administration in a mouse model of AD. We first determined the effects on the suppressed amyloidosis and apoptotic process in the hippocampus of AD mice. We subsequently examined behavioral changes using the Y-maze, fear conditioning, and Morris water maze tests in probiotics treated AD mice. Importantly, we found that treatment with *B. bifidum* BGN4 and *B. longum* BORI significantly improved the cognitive and memory ability of 5xFAD mice, indicating that the administration of probiotic *B. bifidum* BGN4 and *B. longum* BORI in gut microbiota can effectively ameliorate the pathological features of AD.

## Materials and Methods

### Animals and Treatment Protocol

Five familial AD mutations (APP K670N/M671L, V717I, I716V, and PS1 harboring two FAD mutations, M146L and L286V)-expressing 5xFAD transgenic mice were obtained from The Jackson Laboratory (Bar Harbor, ME). Age- and sex-matched C57BI/6 and 5xFAD mice were used for all experiments and randomly assigned to each group. The mice were assigned into four groups: Control-BGN4/BORI group (*n* = 10), Control+BGN4/BORI group (*n* = 10), 5xFAD-BGN4/BORI group (*n* = 10), and 5xFAD+BGN4/BORI group (*n* = 10). For probiotic treatment, freeze dried *B. bifidum* BGN4 and *B. longum* BORI powder (BIFIDO, Gangwon, Korea) were orally administrated to 3-month-old mice daily *via* oral gavage (1 × 10^9^ CFU in 0.2 ml sterile water) for 30 days. All experiments were performed in accordance with the institutional guidelines for animal use and received ethical approval from the Institutional Animal Care and Use Committee at Dongguk University.

### Genomic DNA Extraction

To collect murine fecal samples, each group was placed in a separate sterile cage. The mice were assigned into four groups: Control-BGN4/BORI group, Control+BGN4/BORI group, 5xFAD-BGN4/BORI group, and 5xFAD+BGN4/BORI group. Fecal pellets (for *n* = 3 per group) were collected directly from the anal orifices once a week for 5 weeks and stored at −80°C for analyses. Total bacterial DNA was isolated from the stool by using ZymoBIOMICS™ DNA Miniprep Kit (D4304, Zymo Research Corporation, Irvine, USA). The total DNA was isolated and purified following the manufacturer’s protocol. After extraction, the DNA purity and concentration were measured with a spectrophotometer, Nano-Drop ND-2000 (Thermo Scientific, Waltham, MA) and Qubit 3.0 fluorometer (Thermo Scientific). Samples were stored at −20°C until further analysis.

### Amplification of 16S rRNA Gene and Sequencing

16S rRNA gene amplification and index PCR were conducted following the Illumina 16S Metagenomic Sequencing Library preparation guide (Illumina, San Diego, CA, USA). The 16S sequence was amplified using forward primer and reverse primer. PCR was initially performed using the primer set, MiSeq 341F (5′-TCGTCGGCAGCGTCAG ATGTGTATAAGAGACAGCCTACGGGNGGCWGCAG-3′) and MiSeq 805R (5′-GTCTCGTGGGCTCGGAGATGTGTATAAGAGACAGGACTACHVGGGTATCTAATCC-3′) using 2× Kapa HiFi Hot start Ready Mix DNA polymerase (Kapa Biosystems, Wilmington, MA, USA). PCR products were purified using the Agencourt AMPure XP kit (Beckman Coulter, Brea, CA, USA). Amplification was performed at 95°C (3 min) with 25 cycles of 95°C (30 s), 55°C (30 s), 72°C (30 s), and a final extension of 72°C (5 min). Quantification and size estimation of the library was carried out on the QIAxcel Advanced using QIAxcel DNA High Resolution Kit (QIAGEN, Hilden, Germany). Sequencing was performed using the Illumina MiSeq System (2 × 250 bp paired-end reads; Illumina, USA).

### Bioinformatic Analysis of Sequencing Data

Microbial sequences were processed using QIIME2 (version 2020.6). Raw sequence reads were demultiplexed using the q2-demux plugin, followed by denoising to remove the sequences with low-quality score using DADA2. All amplicon sequence variants (ASVs) were aligned using the MAFFT and were used to create a rooted phylogenetic tree for phylogenetic diversity analysis with FastTree 2 (Price et al., 2010). ASVs were assigned based on pre-built branches from the 99% SILVA 132 database with a naïve Bayes taxonomy classifier developed for the sklearn classifier (Bokulich et al., 2018). All samples were rarefied to a maximum depth (19,431 reads) that could retain all samples. Faith’s phylogenetic diversity and observed features were calculated to measure microbial diversity. Unweighted and weighted UniFrac distance metrics were used to compare the microbial community structure (Lozupone and Knight, 2005; Lozupone et al., 2007). The non-parametric Kruskal-Wallis test was used to determine significant differences in microbial diversity. To evaluate the difference in community structure, PERMANOVA (with 999 random permutations) was used. The linear discriminant analysis effect size (LEfSe) method and LDA effect size (cut-off ≥3) was used to detect significant differences in comparison to the bacteria abundance (Segata et al., 2011). Correlations between gut microbiota and parameters were calculated by using the Spearman’s rank correlation coefficient in R package.

### Accession Number

The accession number for the gut microbiome data reported in this article is PRJNA731317.

### Behavioral Tests

The Y-maze test was performed 30 days after probiotic administration to assess short-term memory. Spontaneous alternation and the number of alternations were tested using white polyvinyl plastic with three open arms (300 mm deep, 150 mm high, 50 mm wide,) at angles of 120° from each other. Mice were placed at the end of one arm and recorded during the 10-min test period. The maze was washed with 70% ethanol after each trial. Data were acquired using the Y-maze software. The sequence of arm entry and the total number of entries was counted by the software to calculate the alternation ratio.

A contextual fear conditioning test was performed using standard chamber with shock floors (20 × 20 cm) and conducted over 3 days. On the 1st day, each mouse was placed in a fear conditioning chamber for 5 min and exposed to white background noise. On the 2nd day of the conditioning session, mice received a 1-s 1.7 mA electric foot shock at 3 min. The training was repeated twice. Twenty-four hours later, mice were exposed to the chamber for 5 min without an electric foot shock. The freezing behavior of mice was videotaped, and the freezing and exploring in the fear conditioning chamber was calculated using specialized behavioral tracking software (EthoVision).

The Morris water maze was performed to test the acquisition of spatial memory and long-term spatial memory. This test was performed in a circular pool (diameter: 90 cm, height: 45 cm) filled with water. An escape platform was placed below the water surface. The water maze test was conducted over 5 days with three trials per day. Each trial was recorded over a 60 s test period. Twenty-four hours after the last training trial, mice were exposed to a 60 s probe test, in which the platform had been removed from the pool. Performance in the probe trial of the water maze was videotaped. The time spent in each quadrant was analyzed using specialized behavioral tracking software (EthoVision).

### Western Blot Analysis

Mouse brains were lysed in RIPA buffer containing 1% NP-40, 0.5% DOC, 0.1% SDS, and 150 mmol/L NaCl in 50 mmol/L Tris (pH 8.0) supplemented with 1× proteinase inhibitor mixture (GenDepot). After adding 5× SDS loading buffer and boiling for 5 min, the samples were centrifuged for 10 min at 12,000 *g*. The supernatants were electrophoresed on 7.5% sodium dodecyl sulfate-polyacrylamide gel and transferred to 0.2 μm nitrocellulose membranes. The membrane was then probed with the following antibodies: Homer1 (1:1,000, Millipore, ABN37), PSD95 (1:1,000, Cell Signaling, 2507S), BDNF (1:1,000, Invitrogen, OSB00017W), APP c-terminal (1:500, Sigma, A8717), and β-actin (1:1,000, Abfrontier, LF-PA0207).

### Immunofluorescence Staining Analysis

Brain samples of control and 5xFAD mice were sliced to 40 μm with a microtome. Brain sections were washed with 1× phosphate-buffered saline after being fixed in 4% paraformaldehyde in phosphate-buffered saline. Brain sections were immunostained according to standard protocols using the following primary antibodies: ChAT (Millipore, AB144P), NeuN (Millipore, MAB377), MAP2 (Invitrogen, 13-1,500), BDNF (Invitrogen, OSB00017W), β-amyloid 1–42 (Biolegend, 805501), Cleaved-caspase3 (Cell Signaling, 9661S) and appropriate fluorescent secondary antibodies (Invitrogen). Next, brain sections were mounted in Fluoromount-G mounting medium. Representative images were then captured using a confocal laser-scanning microscope (*ZEISS*, LSM800). The number of ChAT-expressing neurons and NeuN-expressing cells was assessed in the hippocampal CA1, CA2, and CA3 region using the automatic cell counter plugin in ImageJ. An investigator blinded to the experimental conditions obtained bilateral counts of ChAT- and NeuN-ir from anatomically matched images to produce an average score.

### Quantitative RT-PCR Analysis

Total RNA for all conditions was extracted from the hippocampus and purified using an RNeasy Kit (QIAGEN) according to the manufacturer’s protocols. The AccuPower RT-PCR PreMix (Bioneer) was used to synthesize cDNA from isolated RNA. Quantitative RT-PCR analysis was performed using Platinum SYBR green qPCR SuperMix (Invitrogen) in a Rotor-Gene Q real-time PCR cycler (QIAGEN) with following conditions: 95°C for 15 min followed by 40 cycles of 95°C for 10 s, 55°C for 15 s, 72°C for 20 s; melting curve from 72°C to 95°C every 0.2°C for 1 s per step. Target gene expression was normalized against the expression of a housekeeping gene, GAPDH, in control and 5xFAD mice administrated with sterile water or *B. bifidum* BGN4 and *B. longum* BORI. The following PCR primers were used: *IL-17* Forward: 5′-CCAGGGAGAGCTTCATCTGT-3′ Reverse: 5′-AGGAAGTCCTTGGCCTCAGT-3′, *IL-1β* Forward: 5′-GGATGAGGACATGAGCAA CCT-3′ Reverse: 5′-AGCTCATATGGGTCCGACAG-3′, *IL-6* Forward: 5′-CCGGAGAG GAGACTTCACAG-3′ Reverse: 5′-CAGAATTGCCATTGCACAAC-3′, *IL-10* Forward: 5′-TGCTGCCTGCTCTTACTGAC-3′ Reverse: 5′-TGGCAACCCAAGTAACC CTT-3′, *NF-kB* Forward: 5′-AGGCTCCTGTGCGTGTCTCC-3′ Reverse: 5′-AGGTCCACTGCG AGGTGAAGG-3′, *COX2* Forward: 5′-CTACAAGACGCCACATCCCC-3′ Reverse: 5′-ATGCGTAGAGAGGGGAGAGC-3′.

### TNF-α and IL-12 Quantification

ELISA was performed using a cytokine ELISA combo kit (KOMA Biotech, K033KIT-02) to detect TNF-α and IL-12 concentrations. Whole blood samples were collected from sterile water or probiotic-treated mouse hearts. Serum samples were separated by centrifugation at 1,000 *g* for 10 min in a refrigerated centrifuge. The collected samples were assessed *via* TNF-α and IL-12 ELISA kits, according to the manufacturer’s instructions.

### Statistical Analysis

Data are expressed as the mean ± SEM of three independent experiments. One-way analysis of variance (ANOVA) followed by Tukey–Kramer multiple comparisons test was performed with GraphPad Prism (La Kolla, California, USA). *N* values represent the number of independent experiments, number of individual experiments, or mice. All statistical details of experiments can be found in the figure legends.

## Results

### *B. bifidum* BGN4 and *B. longum* BORI Reduced Hippocampal Neuronal Death in 5xFAD Mice

**Figure 1:**
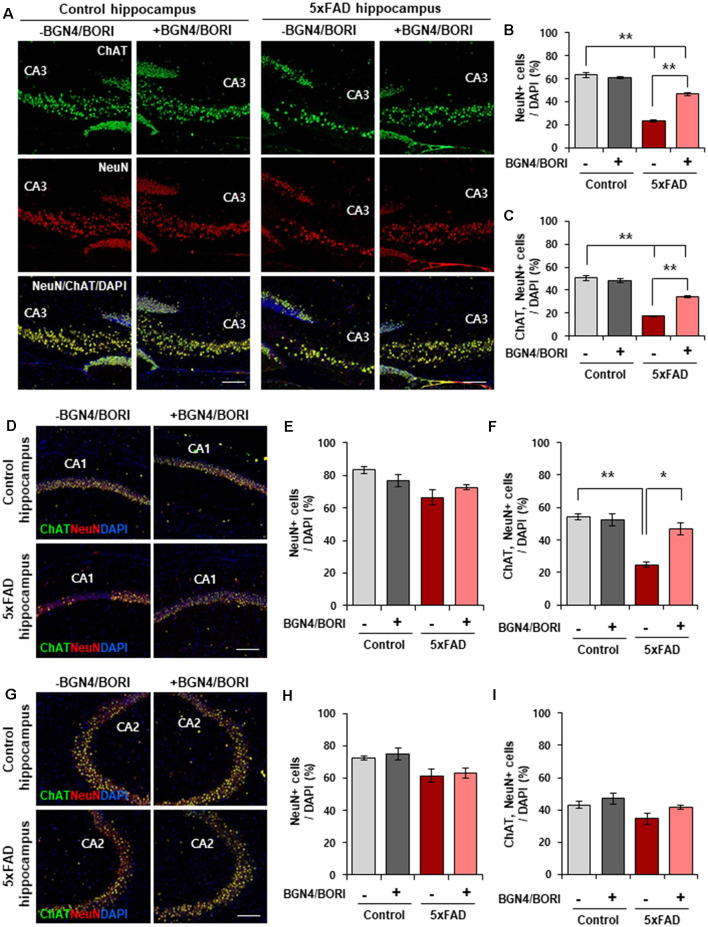
*B. bifidum* BGN4 and *B. longum* BORI reduced hippocampal neuronal death in 5xFAD Mice. **(A)** Immunofluorescence for ChAT and NeuN positive cells in the CA3 hippocampal subfields of control (left) or 5xFAD mice (right) treated with *B. bifidum* BGN4 and *B. longum* BORI (BGN4/BORI). Scale bar = 50 μm. **(B,C)** Quantifications of the NeuN+ **(B)** and ChAT+/NeuN+ **(C)** neurons in the CA3 region at 30 days. Data represent the mean ± SEM. *ANOVA*, **p < 0.01 (*n* = 5). **(D)** Representative images of ChAT and NeuN positive cells in the CA1 hippocampal subfields of control or 5xFAD mice treated *B. bifidum* BGN4 and *B. longum* BORI. Scale bar = 50 μm. **(E,F)** Quantifications of the NeuN+ **(E)** and ChAT+/NeuN+ **(F)** neurons in the CA1 hippocampal region at 30 days. Data represent the mean ± SEM. *ANOVA*, *p < 0.05, **p < 0.01 (*n* = 5). **(G)** Representative images of ChAT and NeuN positive cells in the CA2 hippocampal subfields of control or 5xFAD mice treated *B. bifidum* BGN4 and *B. longum* BORI. Scale bar = 50 μm. **(H,I)** Quantifications of the NeuN+ **(H)** and ChAT+/NeuN+ **(I)** neurons in the CA2 hippocampal region at 30 days. Data represent the mean ± SEM (*n* = 5).

To assess the effect of *B. bifidum* BGN4 and *B. longum* BORI in AD, we first examined the number of hippocampal neuronal cells in the CA3 region of 3 months old probiotic-treated 5xFAD mice. Thirty days after BGN4/BORI treatment (once a day, 1 × 10^9^ CFU in 0.2 ml sterile water), we observed significantly increased NeuN positive cells in the CA3 region of probiotic-treated AD mice hippocampus (Figures 1A,B). Since choline acetyltransferase (ChAT), the enzyme that synthesizes acetylcholine, is expressed during hippocampus-based learning and memory processes (Hawley et al., 2015), we next examined the number of ChAT+ neurons in the AD hippocampus. Consistent with previous results, we observed a significant increase in ChAT+/NeuN+ cells in the CA3 region of probiotic-treated 5xFAD mice hippocampus (Figures 1A,C). Moreover, we confirmed a significant increase in the number of ChAT+/NeuN+ cells in the CA1 region of probiotic-treated 5xFAD mice (Figures 1D–F). However, we observed that the number of NeuN+ and ChAT+/NeuN+ cells was not changed in the CA2 region between groups (Figures 1G–I). These data suggest that *B. bifidum* BGN4 and *B. longum* BORI attenuated hippocampal neuronal death in CA3 and CA1 regions of AD mice.

### *B. bifidum* BGN4 and *B. longum* BORI Restored BDNF and Synaptic Scaffolding Proteins in the Hippocampus of 5xFAD Mice

**Figure 2:**
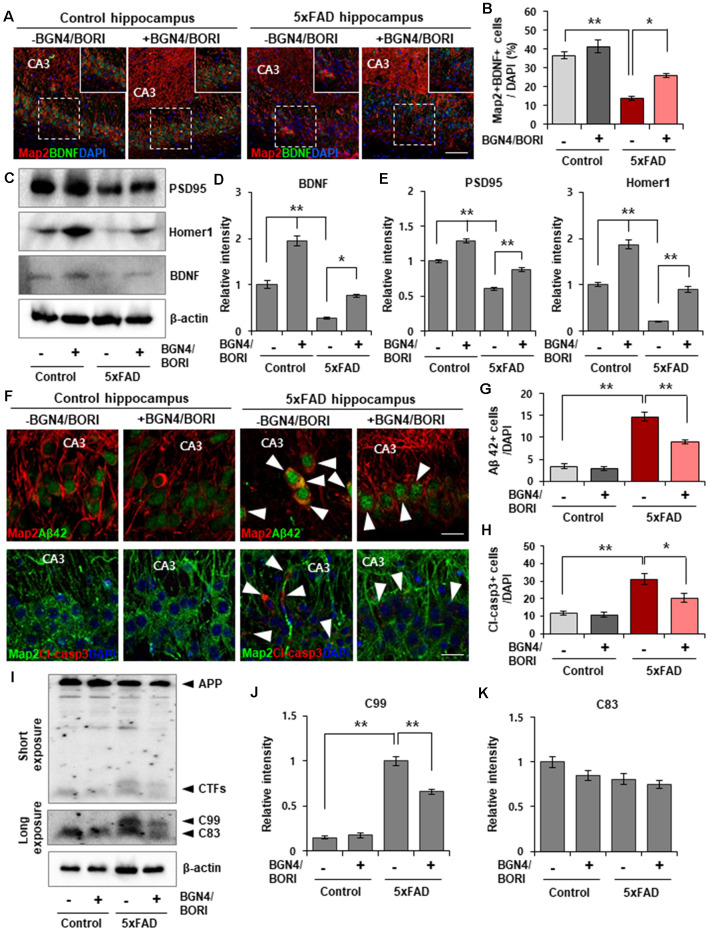
*B. bifidum* BGN4 and *B. longum* BORI reduced Alzheimer’s disease (AD) pathogenesis in the 5xFAD Mouse Brain. **(A)** Representative images of Map2 and BDNF positive cells in the CA3 hippocampal subfields of control or 5xFAD mice treated with *B. bifidum* BGN4 and *B. longum* BORI (BGN4/BORI). Scale bar = 50 μm. **(B)** Quantification of the Map2+/BDNF+ neurons in the CA3 hippocampal region at 30 days. Data represent the mean ± SEM. *ANOVA*, *p < 0.05, **p < 0.01 (*n* = 5).** (C)** Western blot analysis of the scaffolding proteins, PSD95 and Homer1, and blood brain-derived neurotrophic factor, BDNF, in the hippocampus of control or 5xFAD mice treated with *B. bifidum* BGN4 and *B. longum* BORI. **(D)** Hippocampal quantification of BDNF expression were normalized to β-actin. Data represent the mean ± SEM. *ANOVA*, *p < 0.05, **p < 0.01 (*n* = 5). **(E)** Hippocampal quantifications of PSD95 (left) and Homer1 (right) expression were normalized to β-actin. Data represent the mean ± SEM. *ANOVA*, **p < 0.01 (*n* = 5). **(F)** Representative images showing the production of amyloid-β42 (top) and cleaved-caspase3 (bottom) in the CA3 hippocampal subfields of control or 5xFAD mice treated with *B. bifidum* BGN4 and *B. longum* BORI. Scale bar = 20 μm. **(G,H)** Quantifications of the amyloid-β42+ **(G)** and cleaved-caspase3+ **(H)** cells in the CA3 hippocampal region at 30 days. Data represent the mean ± SEM. *ANOVA*, *p < 0.05, **p < 0.01 (*n* = 6). **(I)** Immunodetection of amyloid precursor protein (APP), C99, C83 in Western blots with protein lysates derived from the hippocampus. **(J,K)** Hippocampal quantifications of C99 **(J)** and C83 **(K)** expression were normalized to β-actin. Data represent the mean ± SEM. *ANOVA*, **p < 0.01 (*n* = 5).

To evaluate the effect of* B. bifidum* BGN4 and *B. longum* BORI on functional synaptic plasticity, we next examined the expression level of BDNF, which regulates an important role in hippocampal synaptic plasticity and cognition (Vaynman et al., 2004). We found a significant increase in the number of Map2+/BDNF+ neurons in the hippocampus of 5xFAD mice treated with *B. bifidum* BGN4 and *B. longum* BORI when compared with control-treated 5xFAD mice (Figures 2A,B). Semiquantitative Western blotting confirmed the increased protein expression of BDNF in the hippocampus of probiotic-treated 5xFAD mice (Figures 2C,D). Further, the expression of synaptic scaffolding proteins, including PSD95 and Homer1, was significantly restored in probiotic-treated 5xFAD mice (Figures 2C,E), demonstrating that *B. bifidum* BGN4 and *B. longum* BORI improved the molecular composition of postsynaptic machinery in the mouse model of AD.

### *B. bifidum* BGN4 and *B. longum* BORI Ameliorated the AD Phenotypes of 5xFAD Mice

Next, we examined whether treatment of *B. bifidum* BGN4 and *B. longum* BORI can ameliorate the AD-associated phenotypes in the hippocampus of 5xFAD mice. Initially, we found reduced amyloid-β42 positive cells in the hippocampus of AD mice at 4 weeks after the oral administration of probiotics (Figures 2F,G). Additionally, we observed a decrease in cleaved caspase-3 positive cells, which are expressed in the apoptotic process, in probiotic-treated 5xFAD mice (Figures 2F,H). The production of APP-CTFs, C99 and C83, which are cleaved by β- and α-secretase contributes to the accumulation of amyloid aggregates (Checler, 1995; Lauritzen et al., 2012). Detection of C99 and C83 using the APP c-terminal antibody revealed a decrease in C99 protein expression in the hippocampus of probiotic-treated 5xFAD mice (Figures 2I–K). These results indicated that AD pathology was attenuated by *B. bifidum* BGN4 and *B. longum* BORI administration.

**Figure 3:**
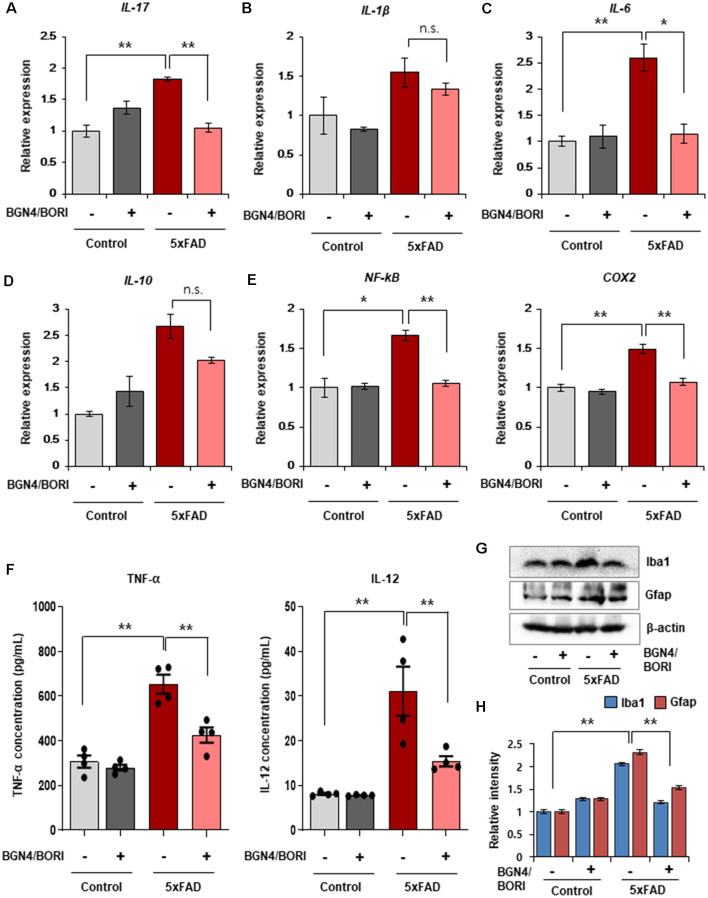
Effects of *B. bifidum* BGN4 and *B. longum* BORI on the neuroinflammatory responses in 5xFAD Mice. **(A–C)** Quantitative RT-PCR analysis of the pro-inflammatory cytokines, *IL-17* **(A)**, *IL-1β* **(B)**, and *IL-6* **(C)** in the hippocampus of control and 5xFAD mice treated with *B. bifidum* BGN4 and *B. longum* BORI (BGN4/BORI). Data represent the mean ± SEM. *ANOVA*, data represent the mean ± SEM. *ANOVA*, *p < 0.05, **p < 0.01; n.s., not significant (*n* = 5). **(D)** Quantitative RT-PCR analysis of the anti-inflammatory cytokine, *IL-10*, in the hippocampus of control or 5xFAD mice treated with *B. bifidum* BGN4 and *B. longum* BORI. Data represent the mean ± SEM, n.s., not significant (*n* = 5). **(E)** Quantitative RT-PCR analysis of inflammation pathway genes, *NF-kB* and *COX2*, in the hippocampus of control or 5xFAD mice treated with *B. bifidum* BGN4 and *B. longum* BORI. Data represent the mean ± SEM. *ANOVA*, *p < 0.05, **p < 0.01 (*n* = 5). **(F)** The concentration of TNF-α and IL-12 in the serum of control or 5xFAD mice treated with *B. bifidum* BGN4 and *B. longum* BORI. Data represent the mean ± SEM. *ANOVA*, **p < 0.01 (*n* = 4). **(G)** Immunodetection of Iba1 and Gfap in Western blots with protein lysates derived from the hippocampus. **(H)** Hippocampal quantification of Iba1 and Gfap expression were normalized to β-actin. Data represent the mean ± SEM. *ANOVA*, **p < 0.01 (*n* = 5).

Next, we investigated the effect of *B. bifidum* BGN4 and *B. longum* BORI on brain inflammatory responses in AD mice. Previous studies have shown that the neuroinflammatory cascade aggravates AD pathogenesis, reduces neuroprotective factors, enhances synaptic dysfunction, and causes neuronal damage, leading to neurodegeneration (Kauwe et al., 2014; Chen et al., 2015). Thus, we determined the expression of inflammatory related genes and cytokines in the probiotic-treated 5xFAD mice. We first observed a statistically marginal increase in the expression of these genes from 5xFAD mice compared to control mice (*n* = 5). Interestingly, *B. bifidum* BGN4 and *B. longum* BORI significantly decreased the expression of *L-17* and *IL-6* in 5xFAD mice, whereas no differences in the expression of *IL-1β* and *IL-10* were observed (Figures 3A–D). To determine how the AD phenotype was ameliorated by *B. bifidum* BGN4 and *B. longum* BORI, we next assessed the expression of the inflammatory pathway factor, *NF-kB*, and its downstream marker, *COX2*. Consistently, *NF-kB* expression was attenuated in *B. bifidum* BGN4 and *B. longum* BORI-treated 5xFAD mice, similar to control levels (Figure 3E, right panel). In addition, we confirmed that the expression of *COX2* was significantly decreased in probiotic-treated 5xFAD mice (Figure 3E, left panel). In the serum, the concentration of the proinflammatory proteins, TNF-α and IL-12, were significantly restored in 5xFAD mice treated with *B. bifidum* BGN4 and *B. longum* BORI (Figure 3F). Finally, we observed that the expression of microglial marker, Iba1, and astrocytic marker, Gfap, were significantly decreased in the hippocampus of probiotic-treated 5xFAD mice compared with control-treated 5xFAD mice (Figures 3G,H). Taken together, these results suggest that *B. bifidum* BGN4 and *B. longum* BORI ameliorates the neuroinflammatory response, which may prevent the progression of the AD phenotype.

### *B. bifidum* BGN4 and *B. longum* BORI Attenuated Cognitive Impairments in 5xFAD Mice

**Figure 4:**
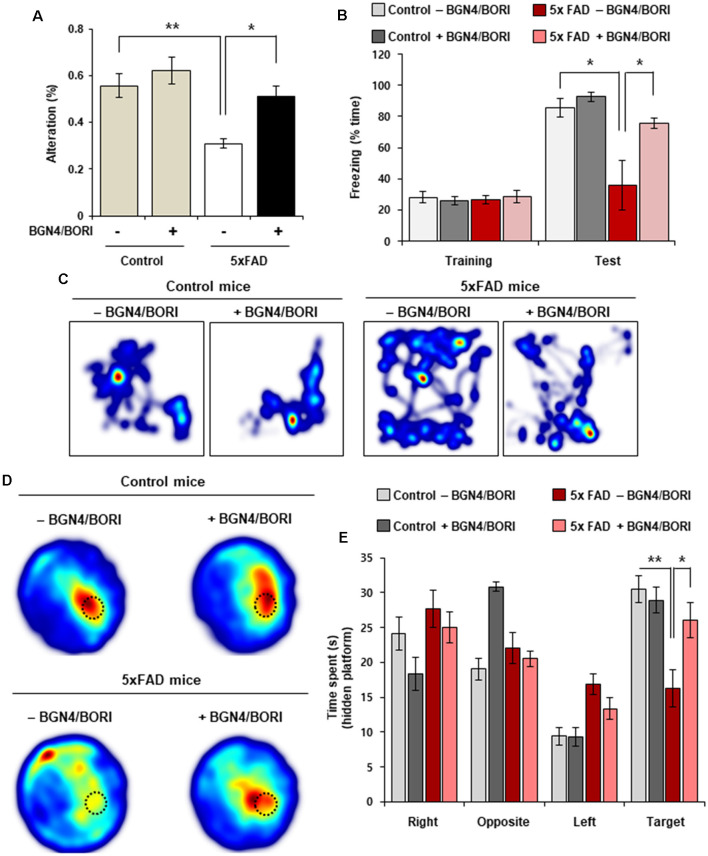
Cognitive impairments was attenuated by *B. bifidum* BGN4 and *B. longum* BORI in 5xFAD Mice. **(A)** Effect of *B. bifidum* BGN4 and *B. longum* BORI (BGN4/BORI) treatment on the performance of control and 5xFAD mice in the Y-maze test. The percent spontaneous alternation was recorded over 5-min in each trial. Data represent the mean ± SEM. *ANOVA*, *p < 0.05, **p < 0.01 (*n* = 6 per group). **(B)** Effect of *B. bifidum* BGN4 and *B. longum* BORI treatment on contextual fear conditioning test. Freezing levels of control (*n* = 5), BGN4/BORI-treated control (*n* = 5), 5xFAD (*n* = 5), and BGN4/BORI-treated 5xFAD (*n* = 6) on day 2. Data represent the mean ± SEM. *ANOVA*, *p < 0.05. **(C)** Representative heatmaps during fear conditioning test. **(D)** Effect of *B. bifidum* BGN4 and *B. longum* BORI on the performance of control and 5xFAD mice in the Morris water maze. Representative heatmaps showing that the time spent in the circular pool to assess long-term spatial learning memory. The dotted circle indicates the target region. **(E)** Quantification of duration in the probe quadrant in the prove trial. Data represent the mean ± SEM. *ANOVA*, *p < 0.05, **p < 0.01 (*n* = 8 per each group).

Next, to investigate whether cognitive deficits can be attenuated by *B. bifidum* BGN4 and *B. longum* BORI treatment, we assessed spatial recognition memory using spontaneous alternation behavior in the Y-maze test. Remarkably, probiotic-treated 5xFAD mice showed an improved alternation performance behavior when compared with non-treated 5xFAD mice (Figure 4A). In the contextual fear conditioning test, we found a significant increase in the freezing ratio in 5xFAD mice treated with *B. bifidum* BGN4 and *B. longum* BORI (Figures 4B,C). Next, we sought to determine whether AD-associated memory loss could be rescued by *B. bifidum* BGN4 and *B. longum* BORI in the Morris water maze test. Importantly, on probe test without a platform, we found that* B. bifidum* BGN4 and *B. longum* BORI treatment dramatically increased time spent in the target quadrant when compared with non-treated 5xFAD mice (Figures 4D,E). These results indicated that *B. bifidum* BGN4 and *B. longum* BORI treatment improved AD-associated memory deficits in 5xFAD mice.

### Altered Gut Microbiome by *B. bifidum* BGN4 and *B. longum* BORI in 5xFAD Mice

**Figure 5:**
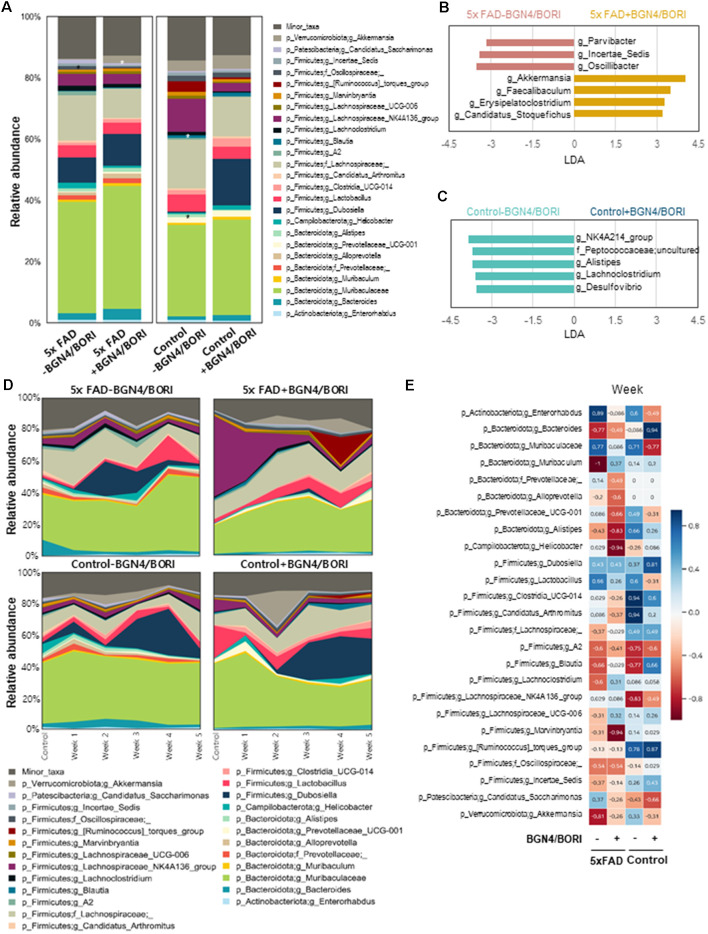
Differences in the gut microbiome following *B. bifidum* BGN4 and *B. longum* BORI Treatment in 5xFAD Mice. **(A)** Comparison of differences in microbial composition between the probiotics intake and the non-intake group (Week 1–5 samples) of 5xFAD or control mice. *Indicates linear discriminant analysis (LDA) score >3.0. Microbial taxa differing **(B)** between 5xFAD and BGN4/BORI-treated 5xFAD mice and **(C)** between control and BGN4/BORI-treated control mice determined by LEfSe analysis (LDA scores >3.0 are shown). **(D)** Changes in microbial composition at genus level in each group over time. **(E)** Spearman’s rank correlation showing the relationship between bacterial abundance at the genus level and time.

To assess the effect of probiotic supplementation on intestinal bacterial communities, mouse fecal microbiota profiles were analyzed during supplementation. To collect murine fecal samples, fecal pellets were collected directly from the anal orifices once a week for 5 weeks in the probiotic treated mice. Interestingly, we found that the 5xFAD mice group fed with *B. bifidum* BGN4 and *B. longum* BORI showed depletions in the bacterial genus *Parvibacter*, *Incertae_Sedis*, and *Oscillibacter*, and enrichments in *Akkermansia*, *Faecalibacterium*, *Erysipelatoclostridium*, and *Candidatus_Stoquefichus* when compared with the control group (LDA >3.0; Figures 5A,B). Moreover, control mice fed with *B. bifidum* BGN4 and *B. longum* BORI showed lower relative abundances of the genus *NK4A214_group*, *Alistipes*, *Lachnoclostridium*, *Desulfovibrio*, and the family Peptococcaceae when compared with non-treated control mice (LDA >3.0; Figures 5A,C). The most relevant change was the enrichment of the genus *Akkermansia*. *Akkermansia* was less abundant in the probiotic-treated 5xFAD mice (0.25%) compared with the untreated 5xFAD mice (1.21%) before the administration of probiotics; this showed a tendency to increase at weeks 1–3 in the 5xFAD mice treated with *B. bifidum* BGN4 and *B. longum* BORI (1.83–5.86%) and decrease in the untreated 5xFAD group (0.15–0.51%; Figure 5D). The relative abundance of *Akkermansia* was strongly correlated with probiotic supplementation periods (ρ = −0.81). In the 5xFAD mice group treated with *B. bifidum* BGN4 and *B. longum* BORI, the marked decline was mitigated, showing a weak negative correlation (ρ = −0.26; Figure 5E). Thus, these results indicated that *B. bifidum* BGN4 and *B. longum* BORI treatment altered Gut Microbiome in 5xFAD mice.

## Discussion

AD is the most common dementia. It is caused by an accumulation of Aβ peptides in the adult brain. Amyloid pathogenesis starts with a nucleation phase that leads to the formation of small aggregates in 2-month-old 5xFAD mice (Oakley et al., 2006). Next, the accumulation of intraneuronal Aβ is rapidly increased during the amyloid growth stage (Takahashi et al., 2002; Eisenberg and Jucker, 2012). Recent studies have identified intestinal microbiome dysbiosis, including decreased Firmicutes, increased Bacteroidetes, and decreased *Bifidobacterium*, in patients with AD (Vogt et al., 2017; Li et al., 2018). These findings suggested that microbiome modulation *via* supplementation of a beneficial and safe probiotic can ameliorate physiological functions, such as the epithelial barrier, gut homeostasis, and the inflammatory response, and improve psychiatric and neurological disease symptoms through the regulation of the gut-brain axis (Ait-Belgnaoui et al., 2014; Akbari et al., 2016). These findings lead us to examine the effects of *B. bifidum* BGN4 and *B. longum* BORI administration on AD pathological features, such as amyloid aggregation, neuroinflammatory response, and AD-associated memory.

In our study, we should note that probiotic supplementation was orally administrated to 3-month-old 5xFAD mice which display early amyloid growth stages; therefore, our data clearly indicate that *B. bifidum* BGN4 and *B. longum* BORI treatment can efficiently reduce amyloid aggregation and cellular apoptosis in the early stages of AD. However, since AD shows a progressive neurodegenerative phenotype, it is important to determine the subsequent effects of this treatment in the middle and late stages of AD. A previous study has demonstrated that treatment with a probiotic mixture containing *Lactobacillus acidophilus*, *Lactobacillus casei*, *Bifidobacterium bifidum*, and *Lactobacillus fermentum* significantly improves the Mini-mental state examination (MMSE) score of patients with severe AD (Akbari et al., 2016). Further studies are required to assess whether probiotic supplementation attenuates the progression of the AD phenotype during the mid and late amyloid-seeding stages.

Moreover, we found that 5xFAD mice group treated with *B. bifidum* BGN4 and* B. longum* BORI, depleted bacterial from the genus, *Parvibacter*, *Incertae_Sedis*, and *Oscillibacter*, and enriched *Bifidobacterium, Akkermansia*, *Faecalibacterium*, *Erysipelatoclostridium*, and *Candidatus_Stoquefichus*, when compared with untreated 5xFAD mice. Among them, the most relevant change was the enrichment of the genus *Akkermansia* and *Faecalibacterium*. Both bacteria possess anti-inflammatory properties (Thursby and Juge, 2017). Interestingly, supplementation of *Akkermansia muciniphila* reduces Aβ 40–42 in the cerebral cortex of APP/PS1 mice, shortens study time, and improves completion rate in Y-maze tests (Ou et al., 2020). Further, this treatment improves intestinal barrier function. Another interesting genus that was enriched was *Faecalibacterium*. This is a representative butyrate-producing bacteria; this substance acts as an anti-inflammatory agent by suppressing the nuclear factor kappa-light-chain-enhancer of the signaling pathways of activated B cells (Schwab et al., 2007). Butyrate drives microglial maturation and is required for the maintenance of mature microglia (Cresci and Bawden, 2015). However, fecal microbiota may not be fully representative of those in the contents or mucosa of the gastrointestinal (GI) tract (Lyra et al., 2012; Lavelle et al., 2015). Thus, the expression of microbial population in fecal samples might be quite different to the intestinal population, which has the capacity to induce changes in the brain. Nevertheless, because of the convenience and non-invasiveness of fecal sampling, many studies have used fecal samples as a proxy to study the gut microbiota. Therefore, a comprehensive understanding of microbial populations between fecal and GI microbiota would help improve longitudinal analyses of microbiota and the application of fecal samples (Lo Presti et al., 2019).

We also explored the neuroinflammatory response that contributes to the progression of AD pathology. Our results showed that probiotic treatment mitigated neuronal inflammation and elevated BDNF expression in AD mice. Previous studies have demonstrated that activated microglia cells were accompanied by increased pro-inflammatory cytokines, such as interleukins and TNF-α, in the AD brain (Vukic et al., 2009; Wang et al., 2015). However, these activated microglia switch to the anti-inflammatory M1-like phenotype which secrete anti-inflammatory cytokines, such as interleukin-4 and interleukin-10, and BDNF that are responsible for inhibiting the innate and adaptive immune reaction and restore synaptic function (Sánchez-Sarasúa et al., 2020). Consistent with this, elevating BDNF expression in the hippocampus of 5xFAD mice was associated with improved cognition (Choi et al., 2018). Thus, our results support that treatment of the *B. bifidum* BGN4 and *B. longum* BORI effectively improved the cognitive functions through an increased BDNF and a decreased neuroinflammatory response in the AD mice.

Moreover, we showed the increase in the synaptic plasticity by oral administration of the *B. bifidum* BGN4 and *B. longum* BORI in control mice (Figures 2C–E), suggesting that altered gut microbiota can modulate the synaptic function through the gut-brain axis even in the control healthy mice. Since increased synaptic plasticity by oral administration of the *B. bifidum* BGN4 and *B. longum* BORI was not previously reported in control mice, our findings provide important implications for the neurotherapeutic effect of the *B. bifidum* BGN4 and *B. longum* BORI.

Taken together, our results demonstrated that treatment with *B. bifidum* BGN4 and *B. longum* BORI ameliorated cognitive dysfunction and memory loss in an AD mouse model *via* elevated BDNF expression. These results indicate the therapeutic potential of *B. bifidum* BGN4 and *B. longum* BORI in preventing the pathological features of AD.

## Conclusion

Our data provide evidence for a therapeutic potential of *B. bifidum* BGN4 and *B. longum* BORI in the mouse model of AD. Administration of *B. bifidum* BGN4 and *B. longum* BORI effectively improves cognition and memory through an increased BDNF and a decreased neuroinflammatory response in the mouse AD hippocampus. Thus, these results indicated that oral treatment with *B. bifidum* BGN4 and *B. longum* BORI could be a novel therapeutic for AD. In the future, it is necessary to verify the efficacy of *B. bifidum* BGN4 and *B. longum* BORI in humans for clinical applications.

## Data Availability Statement

Publicly available datasets were analyzed in this study. This data can be found here: NCBI BioProject, https://www.ncbi.nlm.nih.gov/Traces/study/?acc=PRJNA731317.

## Ethics Statement

The animal study was reviewed and approved by Institutional Animal Care and Use Committee at Dongguk University (IACUC-2021-006-1).

## Author Contributions

HK and SK performed the experiments. HK, S-jP, GP, HS, and MP performed the data analysis. HK, MP, and JK designed the study and contributed to writing the manuscript. All authors contributed to the article and approved the submitted version.

## Conflict of Interest

S-jP and MP are directly employed by Bifido Co., Ltd., and hold BIFIDO Co., Ltd. Stocks as a CTO. The remaining authors declare that the research was conducted in the absence of any commercial or financial relationships that could be construed as a potential conflict of interest.

## Publisher’s Note

All claims expressed in this article are solely those of the authors and do not necessarily represent those of their affiliated organizations, or those of the publisher, the editors and the reviewers. Any product that may be evaluated in this article, or claim that may be made by its manufacturer, is not guaranteed or endorsed by the publisher.
